# Mesenchymal Stromal Cells Accelerate Epithelial Tight Junction Assembly via the AMP-Activated Protein Kinase Pathway, Independently of Liver Kinase B1

**DOI:** 10.1155/2017/9717353

**Published:** 2017-07-11

**Authors:** P. Rowart, P. Erpicum, J.-M. Krzesinski, M. Sebbagh, F. Jouret

**Affiliations:** ^1^Groupe Interdisciplinaire de Génoprotéomique Appliquée (GIGA), Cardiovascular Sciences, University of Liège (ULg), Liège, Belgium; ^2^Division of Nephrology, University of Liège Hospital (ULg CHU), Liège, Belgium; ^3^Centre de Recherche en Cancérologie de Marseille, Aix Marseille Université UM105, Institut Paoli Calmettes, UMR7258 CNRS, U1068 INSERM, Cell Polarity, Cell Signalling and Cancer “Equipe Labellisée Ligue Contre le Cancer”, Marseille, France

## Abstract

**Background:**

Mesenchymal stromal cells (MSC) are fibroblast-like multipotent cells capable of tissue-repair properties. Given the essentiality of tight junctions (TJ) in epithelial integrity, we hypothesized that MSC modulate TJ formation, via the AMP-activated kinase (AMPK) pathway. Liver kinase-*β*1 (LKB1) and Ca^2+^-calmodulin-dependent protein kinase kinase (CaMKK) represent the main kinases that activate AMPK.

**Methods:**

The in vitro Ca^2+^ switch from 5 *μ*M to 1.8 mM was performed using epithelial Madin-Darby canine kidney (MDCK) cells cultured alone or cocultured with rat bone marrow-derived MSC or preexposed to MSC-conditioned medium. TJ assembly was measured by assessing ZO-1 relocation to cell-cell contacts. Experiments were conducted using MDCK stably expressing short-hairpin-RNA (shRNA) against LKB1 or luciferase (LUC, as controls). Compound STO-609 (50 *μ*M) was used as CaMKK inhibitor.

**Results:**

Following Ca^2+^ switch, ZO-1 relocation and phosphorylation/activation of AMPK were significantly higher in MDCK/MSC compared to MDCK. No difference in AMPK phosphorylation was observed between LKB1-shRNA and Luc-shRNA MDCK following Ca^2+^ switch. Conversely, incubation with STO-609 prior to Ca^2+^ switch prevented AMPK phosphorylation and ZO-1 relocation. MSC-conditioned medium slightly but significantly increased AMPK activation and accelerated TJ-associated distribution of ZO-1 post Ca^2+^ switch in comparison to regular medium.

**Conclusions:**

MSC modulate the assembly of epithelial TJ, via the CaMKK/AMPK pathway independently of LKB1.

## 1. Introduction

Epithelial tight junctions (TJ) form a seal at the superior pole of the lateral plasma membrane when cells differentiate and acquire polarity [1]. TJ regulate the passage of ions and small molecules through the paracellular pathway [2] and also restrict the diffusion of membrane proteins between the apical and basolateral compartments. TJ are made of at least 40 different proteins including transmembrane proteins, such as claudins and occludins, and adaptor proteins, such as members of the MAGUK (membrane-associated guanylate kinase) family, ZO-1, ZO-2, and ZO-3 [3, 4]. At the time of TJ assembly, ZO-1 and ZO-2 have essential roles in both organizing TJ components and targeting them to their proper location [5]. Many factors have been identified as modulators of TJ assembly/disassembly, including extracellular Ca^2+^ [6]. Extracellular Ca^2+^ is essential for both the development of new junctions [7] and the stabilization of mature junctions [8, 9] between epithelial cells [10]. The dependence of TJ assembly on Ca^2+^ is probably attributable to the stabilization of the cell adhesion molecule E-cadherin in its adhesive state. Numerous pathways have been implicated in TJ regulation, including the AMP-activated protein kinase [1, 11–16].

AMPK is an ubiquitous heterotrimeric complex made of 1 catalytic *α*-subunit and 2 regulatory *β*- and *γ*-subunits [17]. AMPK activity is modulated by the intracellular AMP-to-ATP ratio, as well as by the activity of upstream AMPK kinases, such as liver kinase-*β*1 (LKB1) and Ca^2+^-calmodulin-dependent protein kinase kinase (CaMKK) [18–20]. An increased ratio of AMP/ATP induces AMP binding to the *γ*-subunit, thereby promoting AMPK phosphorylation at a threonine residue (Thr-172) and its activation [21]. Additionally, in case of energy stress, LKB1 phosphorylates and activates AMPK via the formation of a complex with the pseudokinase STRAD and the scaffolding protein MO25 [22]. CaMKK activates AMPK in an AMP-independent manner in response to increased cytosolic calcium concentration [23, 24]. Note that AMPK auto-phosphorylation at *β*-subunit Thr-148 has been reported [25]. Activated AMPK promotes ATP production by favoring catabolism and switching off anabolic pathways. Interestingly, the pharmacological activation of AMPK by AICAR induces TJ assembly, independently of extracellular Ca^2+^ or energy deprivation [11, 18]. This effect might be achieved by strengthening the *trans* interactions mediated by cell adhesion molecules involved in the nectin–I-afadin system [12, 26] and/or by modulating cytoskeleton dynamics near the cell membrane [12]. Furthermore, preactivation of AMPK by metformin or AICAR helps preserve the functional integrity of epithelial cells in the face of ischemia and energy depletion, as demonstrated in vitro and in vivo [27–29]. TJ disruption is indeed considered as one of the earliest hallmarks of epithelial injury, leading to the loss of cell polarity and tissue disorganization.

Cumulative evidence in the field of epithelial injury supports that mesenchymal stromal cells (MSC) are capable of tissue-repair properties [30]. MSC represent a heterogeneous population of adult fibroblast-like multipotent cells [31]. In addition to their beneficial immunomodulatory and anti-inflammatory abilities [32], MSC may help epithelial cells survive, proliferate, and differentiate following injury [30, 33–35]. Hence, recent in vitro observations highlighted the role of MSC in wound healing of airway epithelium, via direct cell-cell contacts and paracrine activation of the epidermal growth factor receptor [36–38]. Also, MSC are known to release membrane vesicles (MVs) of various size and composition into the extracellular environment [39]. MSC-derived MVs may help transfer cytosolic components, including proteins, lipids, RNA, and organelles, from MSC to neighboring cells, which accelerate tissue repair [40, 41].

In the present study, we first investigated which AMPK kinases were responsible for AMPK phosphorylation and activation at the time of a Ca^2+^ switch. Next, we questioned the impact of MSC on epithelial TJ regulation in a coculture system of Ca^2+^-induced TJ assembly in MDCK cells. Finally, we studied the impact of MSC-conditioned medium on epithelial TJ assembly. This paper was presented at MiSOT 2016—The 6th Expert Meeting on Therapeutic MSCs for Immune Modulation.

## 2. Materials and Methods

### 2.1. MDCK Culture Conditions

MDCK cells were grown to confluence in *α*-MEM supplemented with 10% FBS, 1% L-glutamine (Lonza), and 1% penicillin (Lonza), in a humidified atmosphere containing 5% CO_2_ at 37°C. MDCK shRNA for *LKB1* and *luciferase* (*Luc,* used as controls) were generated using pSUPER/retro-puro vector, as previously reported [24]. Stable populations were maintained using puromycin (2 *μ*g/mL; Sigma) as selection agent.

### 2.2. Isolation and Characterization of Bone Marrow-Derived MSC

Bone marrow cells were flushed from both femurs and tibias of male 9-week-old *Lewis* rats using phosphate-buffered saline (PBS, Lonza). After homogenization, cell suspension was filtered and centrifuged at 1200 rpm for 10 min. Cells were resuspended in *α*-MEM medium (Lonza) and gently sieved through Ficoll (Healthcare Life Sciences). After an additional 1500 rpm centrifugation for 45 min at room temperature (RT), mononuclear cells were removed from the gradient interface and suspended in *α*-MEM solution before final 1200 rpm centrifugation for 10 min. The cells were then plated in 75 cm^2^ culture flask containing *α*-MEM supplemented with 10% FBS, 1% L-glutamine, and 1% penicillin. MSC were maintained at 37°C in a humidified 5% CO_2_ incubator. Supplemented *α*-MEM was changed twice a week. Cells were trypsinised at 80% of confluence for maximum 8 passages. At confluence, fresh culture medium was poured, collected at day 3, centrifuged at 1.800 rpm for 5 min, and stored at −80°C for further use. MSC phenotype was tested according to the criteria of the International Society of Cell Therapy: (i) plastic adherence, (ii) (non)expression of conventional surface markers using flow cytometry; and (iii) differentiation into adipogenic, osteogenic, and chondrogenic lineages [42]. Flow cytometry was performed on a FACSCalibur flow cytometer (BD Biosciences), using Alexa Fluor-conjugated anti-rat CD29 antibody (BD Pharmingen), APC-conjugated anti-rat CD90 antibody (BD Pharmingen), V450-conjugated anti-rat CD45 antibody (BD Horizon), FITC-conjugated anti-rat CD11b (BD Pharmingen), and PE-conjugated anti-CD79a antibody (Abcam).

### 2.3. MSC/MDCK Coculture System

Cell populations were mixed and seeded on 6-well plates at density 1.5 × 10^5^ cells/well. The seeding ratio of MSC : MDCK was 1 : 3. All experiments were performed at confluence. Alternatively, MDCK cells were incubated with 2 mL of *α*-MEM medium preexposed to MSC for 3 days.

### 2.4. Ca^2+^ Switch Experiments

Following steady state, cells were rinsed and incubated in Ca^2+^-free S-MEM supplemented with 5% dialyzed FBS ([Ca^2+^], 5 *μ*M) for 16 h before being switched back to normal medium (*α*-MEM; [Ca^2+^], 1.8 mM) for the indicated times. Compounds STO-609 (50 *μ*M; Sigma) and dorsomorphin/compound C (50 *μ*M; Sigma) were used as CaMKK and AMPK inhibitors, respectively. Increasing concentrations of dorsomorphin/compound C were tested in order to fully block AMPK activation (Supplementary Figure, panel A, available online at https://doi.org/10.1155/2017/9717353). Alternatively, MDCK cells were exposed to MSC-conditioned medium for 24 h, before exposure to S-MEM for 16 h. The Ca^2+^ switch was then realized using MSC-conditioned *α*-MEM for the indicated times.

### 2.5. Western Blot Analysis

Cells were lysed on ice in RIPA lysis buffer including protease and phosphatase inhibitors (Roche). Cell lysates were obtained by centrifugation at 13.000 rpm for 30 min at 4°C. Supernatant was collected. Protein concentration was determined using Bradford method. Protein lysates were mixed with Laemmli buffer (1 : 4) and heated for 2 min at 95°C. Equal amounts of protein (30 *μ*g/lane) were loaded onto stain-free SDS electrophoresis gels and separated at 100 V (Bio-Rad). Gels were exposed to UV light for 5 min (ChemiDoc MP system, Bio-Rad). Proteins were transferred to PVDF membranes (previously activated by ethanol) using the Trans-Blot Turbo transfer system for 7 min at RT. Blots were blocked with 5% milk in Tris-buffered saline with Tween 20 (TBS-T) for 1 h and incubated overnight at 4°C with primary antibodies: pAMPK (T172) (Cell Signaling), AMPK (Cell Signaling), and pACC (Cell Signaling) antibodies. Blots were rinsing 5 times with TBS-T for 5 min and incubated with HRP-conjugated anti-rabbit secondary antibodies (1/4000) for 90 min at RT. After rinsing, chemiluminescent signals were captured by ChemiDoc MP system after applying chemiluminescent substrate (Femto, Thermoscientific) on blots. Image data were analyzed and quantified (*n* = 4 for each experimental condition) using Image Lab 4.1 software. Representative samples were then run on the same stain-free SDS gels for the sake of publication, in agreement with the ASBMB policy.

### 2.6. Immunofluorescence and Quantification of ZO-1 Deposits

Cells grown on coverslips were rinsed twice with PBS and fixed in cold methanol for 12 min. After blockade with PBS/BSA 5% dilution for 60 min at RT and incubated for 90 min with anti-ZO-1 (ThermoFisher Scientific) and followed by 60 min of incubation with Alexa Fluor 488-conjugated anti-rabbit IgG (Molecular Probes), cells were visualized on an FSX-100 (Olympus Life Science). Contrast, brightness, and focus settings were chosen so that all pixels were in the linear range. To quantify the average ZO-1 length per cell, 4 fields were randomly selected, and the total length of ZO-1 in each field was outlined manually on Photoshop, followed by measurement using Image J software (NIH) [11–13]. Cell numbers were counted for each field with the DAPI Fluoromount-G (SouthernBiotech) slide mounting.

### 2.7. Statistical Analyses

Data were expressed as mean ± 1 standard deviation (SD). One-way analysis of variance, Mann–Whitney, and Student *t*-test were appropriately performed, with a significant *p* value set at 0.05 (MedCalc software).

## 3. Results

### 3.1. The Phosphorylation and Activation of AMPK Following a Ca^2+^ Switch Depend on CaMKK in MDCK Cells, Independently of LKB1

**Figure 1:**
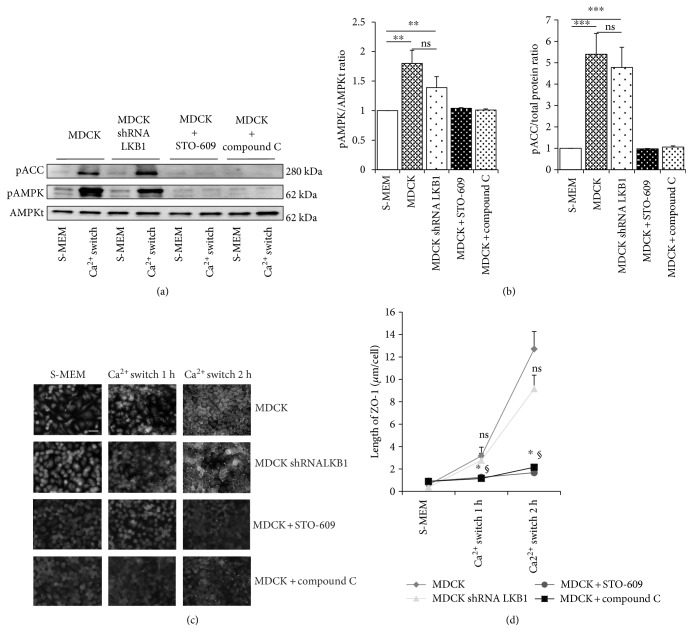
Role of the AMPK kinases, LKB1 and CaMKK, in AMPK activation and ZO-1 relocation following a Ca^2+^ switch in MDCK cells. Representative immunoblotting (a) and quantifications (b) of phospho-acetyl-Coa carboxylase (pACC), phospho-AMP-activated protein kinase (pAMPK), and total AMPK (AMPKt) in low Ca^2+^ conditions (S-MEM) and following Ca^2+^ switch using MDCK cells or LKB1-shRNA MDCK cells. Compounds STO-609 and C were used as CaMKK and AMPK inhibitors, respectively. Quantifications of immunoreactive signals were performed by stain-free method after normalization to total protein content of each lane. Quantifications of phospho-ACC, phospho-AMPK, and AMPKt signals following Ca^2+^ switch were calculated and expressed by the ratio to the immunoreactive signal of SMEM condition in each individual experiment (a). For the sake of bar-graph clarity (b), SMEM values of all experiments were normalized to 1 in order to represent mean ratios of phospho-ACC/total protein content and phospho-AMPK/AMPKt in different experimental conditions (b). Data are presented as mean ± SD; ns: not significant, ^∗∗^*p* ≤ 0.01, ^∗∗∗^*p* ≤ 0.001. Representative immunofluorescence (c) and quantifications (d) of ZO-1 deposits at increasing time points following Ca^2+^ switch in similar conditions as in (a) and (b) (scale bar: 16 *μ*m). No statistically significant difference was observed between MDCK and LKB1-shRNA MDCK (ns: not significant). MDCK exposed to compound C (^∗^*p* ≤ 0.01) or STO-609 (^§^*p* ≤ 0.01) showed a significant reduction of ZO-1 relocation in comparison to control MDCK. Data are presented as mean ± SD.

Following a Ca^2+^ switch, we observed a mean 1.75-fold increase of pAMPK compared to S-MEM medium (*n* = 4, *p* < 0.05) whereas total AMPK remained unchanged, as previously reported [11] (Figures 1(a) and 1(b)). Mean levels of pACC, a typical substrate of AMPK, followed a similar pattern, with a 5.3-fold increase following Ca^2+^ switch (*n* = 4, *p* < 0.05). LKB1 and CaMKK are considered as the 2 major AMPK kinases [23]. In LKB1-shRNA MDCK cells, mean levels of pAMPK and pACC were 1.4-fold and 4.7-fold increase, respectively, in comparison to S-MEM (*n* = 4, *p* < 0.05), with no significant difference with control Luc-shRNA (*n* = 4, not significant (ns)) (Figures 1(a) and 1(b)). Of important note, Luc-shRNA MDCK cells behave similarly as MDCK cells regarding AMPK phosphorylation/activation and ZO-1 relocation following Ca^2+^ switch (*n* = 4, data not shown). Conversely, pharmacological inhibition of CaMKK using STO-609 prevented AMPK phosphorylation and activation after Ca^2+^ switch, with mean levels of pAMPK and pACC similar to S-MEM conditions (*n* = 4, ns). Incubation of MDCK with AMPK inhibitor, dorsomorphin/compound C (50 *μ*M) prevented AMPK autophosphorylation classically induced by Ca^2+^ switch (Figures 1(a) and 1(b)). These observations suggest that CaMKK plays a role in Ca^2+^-induced AMPK activation in MDCK cells, independently of LKB1.

### 3.2. Pharmacological Inhibitions of AMPK or CaMKK Prevent Ca^2+^-Induced TJ Relocation of ZO-1

During a Ca^2+^ switch, the translocation of TJ-associated protein ZO-1 from cell cytosol to cell-cell junctions represents a key and early step of TJ assembly [5]. Hence, we monitored the length of ZO-1 membrane deposits following a Ca^2+^ switch in MDCK cells exposed to various experimental conditions [11–13]. In normal conditions, readdition of Ca^2+^ causes a 4-fold increase of ZO-1 length compared to S-MEM conditions (Figures 1(c) and 1(d)). After 2 h of Ca^2+^ switch, TJ were largely assembled as a classical chicken-wire network. In LKB1-shRNA MDCK cells, ZO-1 relocation followed a pattern similar to control MDCK (*n* = 4, *not significant*). By contrast, pharmacological inhibition of CaMKK (using STO-609) (*n* = 4, *p* < 0.05) or AMPK (dorsomorphin/compound C) (*n* = 4, *p* < 0.05) prevented ZO-1 relocation induced by the Ca^2+^ switch (Figures 1(c) and 1(d)). These observations suggest that AMPK and CaMKK kinase activity participates in Ca^2+^-induced ZO-1 deposits in MDCK cells, independently of LKB1.

### 3.3. The Phosphorylation and Activation of AMPK in MDCK Cells Are Enhanced in the Presence of MSC, Which Is Associated with Faster Relocation of ZO-1 to Cell-Cell Contacts

**Figure 2:**
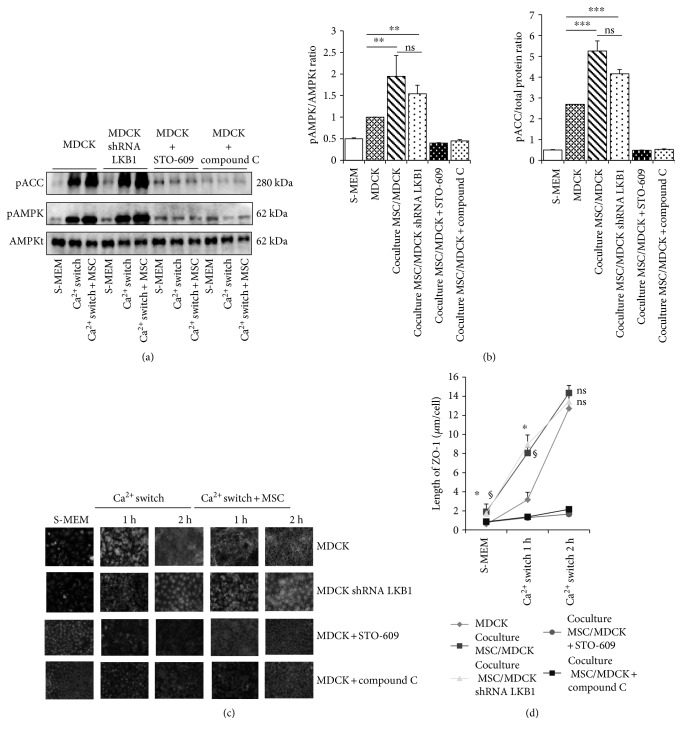
Impact of mesenchymal stromal cells (MSC) on AMPK activation and ZO-1 relocation following a Ca^2+^ switch in MDCK cells. Representative immunoblotting (a) and quantifications (b) of phospho-acetyl-Coa carboxylase (pACC), phospho-AMP-activated protein kinase (pAMPK), and total AMPK (AMPKt) in low Ca^2+^ conditions (S-MEM) and following Ca^2+^ switch using MDCK cells or LKB1-shRNA MDCK cells, with versus without MSC. Compounds STO-609 and C were used as CaMKK and AMPK inhibitors, respectively. Quantifications of immunoreactive signals were performed by stain-free method after normalization to total protein content of each lane. Data are presented as mean ± SD; ns: not significant, ^∗∗^*p* ≤ 0.01, ^∗∗∗^*p* ≤ 0.001. Representative immunofluorescence (c) and quantifications (d) of ZO-1 deposits at increasing time points following Ca^2+^ switch in similar conditions as in (a) and (b) (scale bar: 16 *μ*m). MSC/MDCK (i.e., MDCK (^§^*p* ≤ 0.01) or MDCK LKB1-shRNA (^∗^*p* ≤ 0.01)) cocultures show significantly increased ZO-1 deposits at 1-hour post Ca^2+^ switch in comparison to MDCK alone. At 2 hours post Ca^2+^ switch, no significant (ns) difference in ZO-1 lengths is observed between MDCK and MSC/MDCKs. Data are presented as mean ± SD.

TJ assembly in epithelial cells may be modulated by nonepithelial cells [43, 44]. As an example, lymphocytes have been shown to boost Ca^2+^-induced activation of AMPK and accelerate TJ formation [43]. Similarly, we postulated that MSC may participate in TJ formation, and we investigated whether AMPK was implicated in such a process. In comparison to MDCK alone, phosphorylation and activation of AMPK was significantly increased in MDCK/MSC coculture, as demonstrated by mean levels of pAMPK/AMPK ratio (*n* = 4, *p* < 0.05) and pACC (*n* = 4, *p* < 0.05) (Figures 2(a) and 2(b)). Of important note, immunoreactive signals for AMPK activation and pACC were undetectable in MSC alone, which suggest that only MDCK AMPK activation pathway is tested in our model [45] (Supplementary Figure, panel B). In line with these observations, we monitored the time-course of ZO-1 relocation to cell-cell contacts following Ca^2+^ switch in the presence or absence of MSC (Figures 2(c) and 2(d)). After 16-hour deprivation of Ca^2+^, the length of TJ-associated ZO-1 per cell was 3x higher in MDCK/MSC coculture compared to MDCK cells alone (*n* = 4, *p* < 0.05) (Figures 2(c) and 2(d)). After 1 h of Ca^2+^ switch, ZO-1 relocation to cell-cell contacts was twice higher in MDCK/MSC than in MDCK alone (*n* = 4, *p* < 0.05). Still, at 2 hours post Ca^2+^ switch, the length of membrane-associated ZO-1 per cell was similar in both groups (*n* = 4, ns) (Figures 2(c) and 2(d)). Of note, immunofluorescence signal for ZO-1 was undetectable in MSC cultured alone, which suggests that ZO-1 quantification only reflects ZO-1 deposits in MDCK cells in our model (Supplementary Figure, panel C). As a whole, these results indicate that MSC may accelerate ZO-1 deposition to cell-cell contacts at the time of TJ assembly in MDCK cells.

### 3.4. MSC-Associated AMPK Activation and ZO-1 Relocation in MDCK Cells Following Ca^2+^ Switch Are Prevented by AMPK and CaMKK Inhibitors Independent of LKB1

Using LKB1-shRNA and Luc-shRNA MDCK cells cocultured with MSC, we assessed the role of LKB1 in MSC-enhanced AMPK activation and ZO-1 relocation. In LKB1-shRNA MDCK cells, mean levels of pAMPK/AMPK ratio (*n* = 4, *p* < 0.05) and pACC (*n* = 4, *p* < 0.05) were, respectively, 1.5-fold increased and 2-fold in the presence versus absence of MSC, to a similar extent of Luc-shRNA MDCK cells (Figures 2(a) and 2(b)). Furthermore, after a 16-hour deprivation of Ca^2+^, the length of ZO-1 remaining at TJ sites per cell was 4x higher in MSC cocultured with LKB1-shRNA MDCK in comparison to MDCK alone (*n* = 4, *p* < 0.05) (Figures 2(c) and 2(d)). After 1 h of Ca^2+^ switch, the length of ZO-1 membrane deposits per cell was twice longer in the presence versus absence of MSC (*n* = 4, *p* < 0.05). In strong contrast, incubation of MSC/MDCK or MDCK cells alone with CaMKK (STO-609) or AMPK (dorsomorphin/compound C) inhibitors prevented MSC impact on AMPK activation and ZO-1 distribution, both after Ca^2+^ deprivation and Ca^2+^ switch (Figures 2(a) and 2(d)). These data suggest that, in a coculture system, MSC modulate Ca^2+^-induced CaMKK-mediated AMPK activation at the time of TJ assembly epithelial cells, independently of LKB1.

### 3.5. MSC-Conditioned Culture Medium Slightly but Significantly Enhances AMPK Activation and ZO-1 Relocation Following Ca^2+^ Switch in MDCK Cells

**Figure 3:**
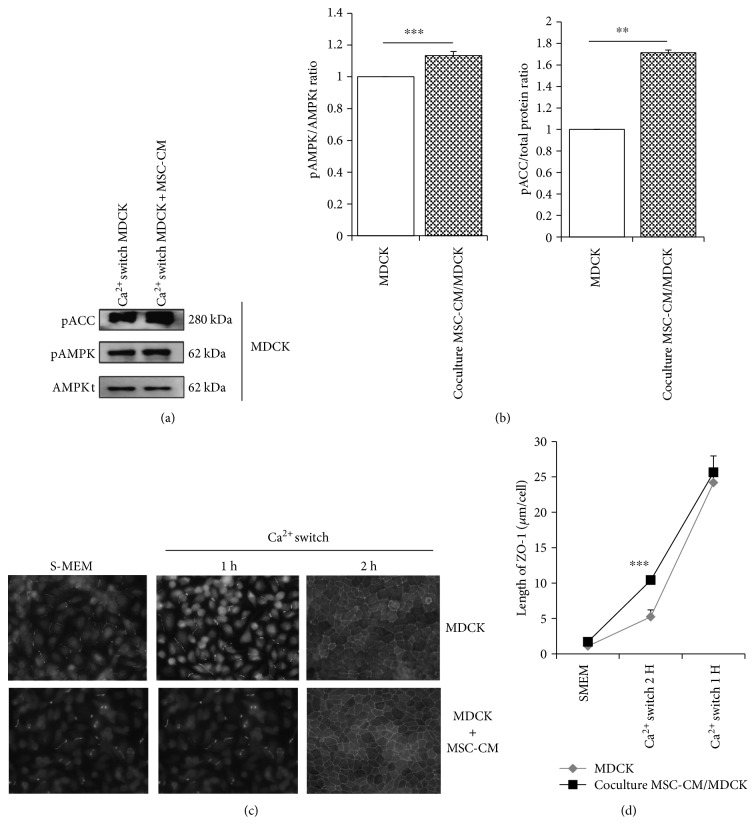
Impact of mesenchymal stromal cell- (MSC-) conditioned medium (CM) on AMPK activation and ZO-1 relocation following a Ca^2+^ switch in MDCK cells. Representative immunoblotting (a) and quantifications (b) of phospho-AMP-activated protein kinase (pAMPK) and total AMPK (AMPKt) in low Ca^2+^ conditions (S-MEM) and following Ca^2+^ switch using MDCK cells exposed to regular versus MSC-preexposed medium. Quantifications of immunoreactive signals were performed by stain-free method after normalization to total protein content of each lane. Representative immunofluorescence (c) and quantifications (d) of ZO-1 deposits at increasing time points following Ca^2+^ switch in similar conditions as in (a) and (b) (scale bar: 16 *μ*m). Data are presented as mean ± SD; ^∗∗∗^*p* ≤ 0.001.

Mechanisms of MSC properties involve both direct cell-cell contacts and indirect impacts via paracrine factors [46]. To assess whether the impact of MSC on Ca^2+^-induced TJ assembly in MDCK cells requires direct cell-cell interactions, we performed the Ca^2+^ switch using an *α*-MEM culture medium preexposed to MSC for 3 days. Hence, we observed that MSC-conditioned medium slightly (1.14-fold) but significantly (*n* = 4, *p* < 0.05) increased AMPK phosphorylation. Thus, pACC was also increased by 1.7-fold in the coculture (*n* = 4, *p* < 0.05). The relocation of ZO-1 was 1.7-fold accelerated at 1-hour post Ca^2+^ switch in comparison to untreated *α*-MEM (*n* = 4, *p* < 0.05) (Figures 3(a) and 3(d)). There was no difference between MSC-conditioned and untreated *α*-MEM at 2 hours post Ca^2+^ switch (Figures 3(b) and 3(c)).

## 4. Discussion

The present in vitro observations suggest that bone marrow-derived MSC modulate epithelial TJ at the time of their Ca^2+^-induced assembly. The relocation of TJ-associated adaptor protein, ZO-1, to MDCK cell-cell contacts was indeed significantly accelerated in the presence of MSC. Furthermore, AMPK phosphorylation and activation at the time of Ca^2+^-induced epithelial TJ assembly were significantly enhanced when MDCK cells were cocultured with MSC, which could be prevented by the pharmacological inhibition of CaMKK. Conversely, the depletion of LKB1 did not significantly influence AMPK phosphorylation following Ca^2+^ switch, with or without MSC coculture.

AMPK activity is modulated by 2 major upstream kinases, that is, LKB1 and CaMKK [18–20, 47]. Still, the respective contribution of each of these AMPK kinases in AMPK activation at the time of a Ca^2+^ switch remains unknown [11, 18]. LKB1 provides a high basal level of AMPK phosphorylation, which is modulated by the binding of AMP to the AMPK *γ*-subunit. AMP binding to the *γ*-subunit allosterically activates AMPK, making it more susceptible for phosphorylation of the *α*-subunit activation loop (at residue Thr172) by LKB1 [48]. Note that AMPK activation associated with Ca^2+^-induced TJ assembly is independent of changes in AMP/ATP ratio or energy privation [11, 18]. Conversely, CaMKK kinase has been shown to trigger AMPK phosphorylation on Thr172 in response to increased intracellular Ca^2+^ concentration with no necessary changes in AMP or ADP levels [21, 49]. Our present in vitro observations further support a role for CaMKK in the activation of AMPK during a Ca^2+^ switch, independently of LKB1 activity. Hence, the pharmacological inhibition of CaMKK hampered AMPK phosphorylation and ZO-1 relocation when culture conditions were shifted from low to high Ca^2+^ concentration, whereas the inactivation of LKB1 did not significantly influence these processes.

Circulating factors and cells have been shown to modulate TJ formation and maintenance in epithelia [50, 51]. Hence, lymphocytes accelerate TJ assembly in a coculture in vitro model compared to epithelial cells alone [43]. This acceleration was found to be mediated by AMPK, independently of changes in cellular ATP levels. Furthermore, it was found to be activated by the proinflammatory cytokine TNF-alpha [43]. In line with these observations, coculturing endometrial epithelial cells with peripheral blood leukocytes improves both the survival of leukocytes and the epithelial barrier function, as reflected by a 4-fold increase in the transepithelial resistance as compared to epithelial cells alone [44]. In this study, direct cell-cell contacts were required for the beneficial impact of immune cells. In our model, we hypothesized that MSC may also influence TJ of epithelial cells given the previous reports about their tissue-repair properties in various organs and tissues [31, 32, 52, 53]. MSC effects are known to be mediated by both direct cell-cell contacts and paracrine secretion of MVs [54, 55]. Using a classical model of Ca^2+^-induced TJ assembly [8], we found that the presence of MSC was associated with a significantly faster deposition of ZO-1 to cell-cell contacts. Furthermore, MSC influence was abrogated in case of cell incubation with CaMKK or AMPK inhibitors, suggesting a key role of the AMPK pathway in such a process. Of important note, AMPK-dependent and independent roles of compound C have been reported in a context-dependent manner [56].

These observations could be partly reproduced by incubating epithelial cells with MSC-conditioned medium, which supports a fractional role for MSC-derived MVs in epithelial TJ regulation.

## 5. Conclusion

As a whole, we report on the role of CaMKK as AMPK kinase at the time of Ca^2+^-induced assembly of epithelial TJ, independently of LKB1. Moreover, we highlight the impact of MSC in the AMPK-mediated regulation of epithelial TJ, via both direct cell-cell contacts and MSC-derivated particles and MSC-derived MVs. These findings open novel research avenues in the deciphering of MSC repair properties.

## Supplementary Material

Supplementary Figure. Panel A. Representative immunoblotting of phospho-AMP-activated protein kinase (pAMPK) and total AMPK (AMPKt) in low-Ca2+ conditions (S-MEM) and following Ca2+ switch using epithelial MDCK cells exposed to [10μM] and [50μM] compound C. Panel B. Representative immunoblotting of phospho-AMP-activated protein kinase (pAMPK) and total AMPK (AMPKt) at baseline (α-MEM), in low-Ca2+ conditions (S-MEM) and following Ca2+ switch using epithelial MDCK cells or mesenchymal stromal cells (MSC) or MDCK/MSC co-cultured cells. Each SDS Page was ran and transferred at the same time and incubated with the same primary and secondary antibodies for the same times. Panel C. Representative immunofluorescence of ZO-1 in MSC cultured alone and MDCK cultured alone. ZO-1 experiments were realized simultaneously using the same reagents for the same time of incubation.
