# Supplementary material for: Mesenchymal Stromal Cells Accelerate Epithelial Tight Junction Assembly via the AMP-Activated Protein Kinase Pathway, Independently of Liver Kinase B1
Source: Stem Cells Int. 2017 Jul 11;2017:9717353. doi: 10.1155/2017/9717353 (PMC5525096; doi:10.1155/2017/9717353)
Supplement: Supplementary file 1 — Supplementary Figure. Panel A. Representative immunoblotting of phospho-AMP-activated protein kinase (pAMPK) and total AMPK (AMPKt) in low-Ca2+ conditions (S-MEM) and following Ca2+ switch using epithelial MDCK cells exposed to [10μM] and [50μM] compound C. Panel B. Representative immunoblotting of phospho-AMP-activated protein kinase (pAMPK) and total AMPK (AMPKt) at baseline (α-MEM), in low-Ca2+ conditions (S-MEM) and following Ca2+ switch using epithelial MDCK cells or mesenchymal stromal cells (MSC) or MDCK/MSC co-cultured cells. Each SDS Page was ran and transferred at the same time and incubated with the same primary and secondary antibodies for the same times. Panel C. Representative immunofluorescence of ZO-1 in MSC cultured alone and MDCK cultured alone. ZO-1 experiments were realized simultaneously using the same reagents for the same time of incubation. [file 9717353.f1.docx]

**MDCK**

**+**

**Compound C (10µM)**

**MDCK**

**+**

**Compound C (50µM)**

**Ca^2+^ Switch**

**SMEM**

**AMPKt**

**pAMPK**

**pACC**

**α-MEM**

**SMEM**

**Ca^2+^ Switch**

**α-MEM**

**SMEM**

**α-MEM**

**Ca^2+^ Switch**

**MSC**

**MDCK**

**Co-culture MSC/MDCK**

**MDCK**

**+**

**DMSO**

A

Supplementary Data

**
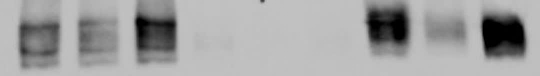

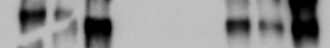

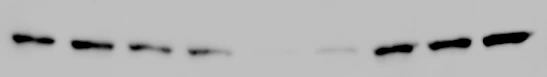
**
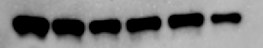

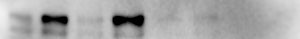

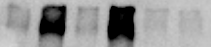
**
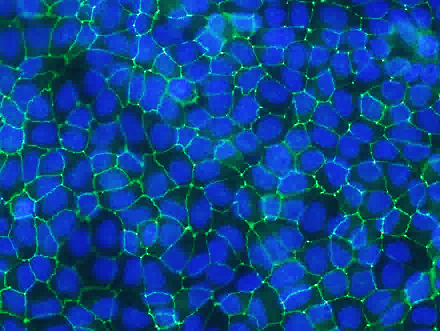

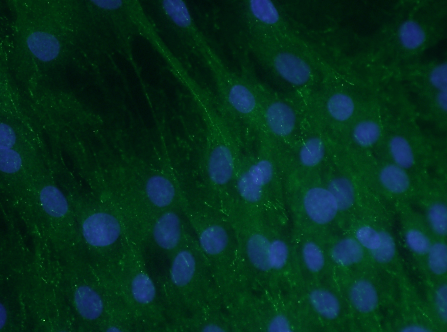
**

*62 kDa*

*62 kDa*

*280 kDa*

*62 kDa*

*62 kDa*

*280 kDa*

**SMEM**

**Ca^2+^ Switch**

**SMEM**

**Ca^2+^ Switch**

**Ca^2+^ Switch**

**SMEM**

**AMPKt**

**pAMPK**

**pACC**

**MDCK**

**α-MEM**

**MSC**

C

B
